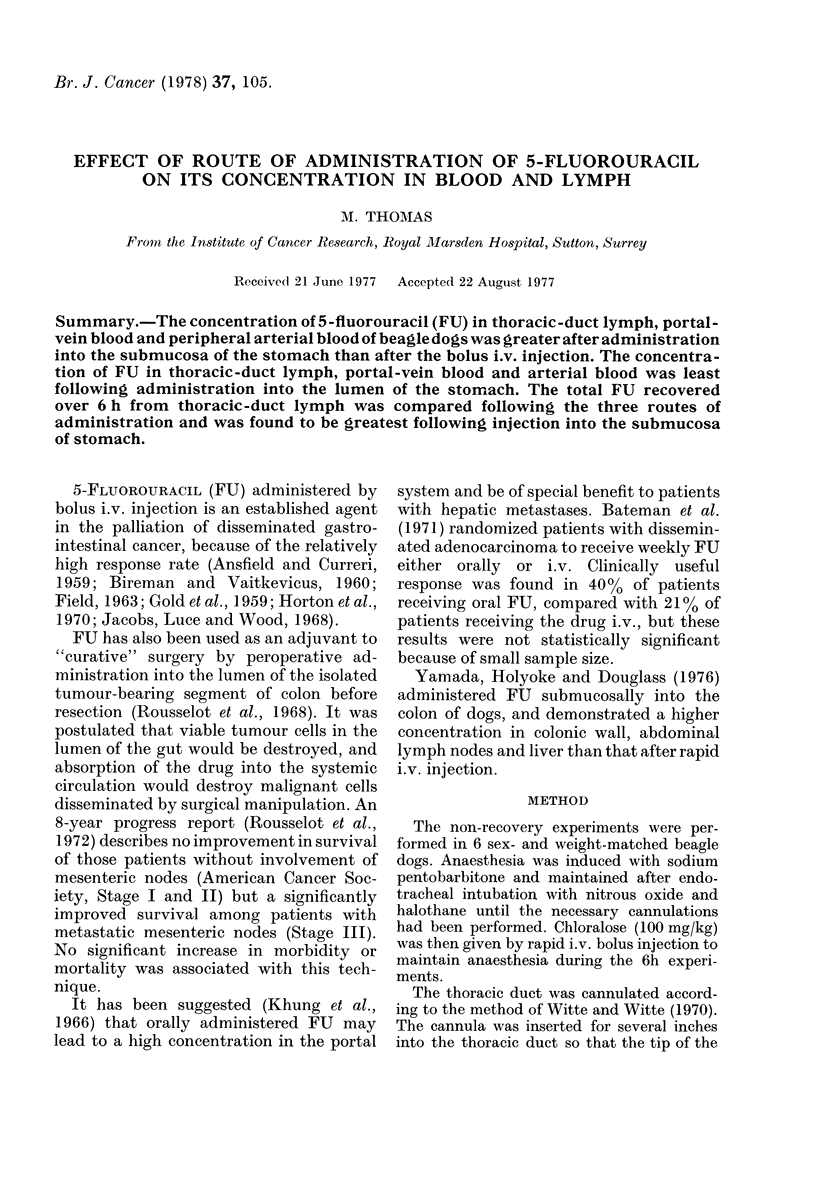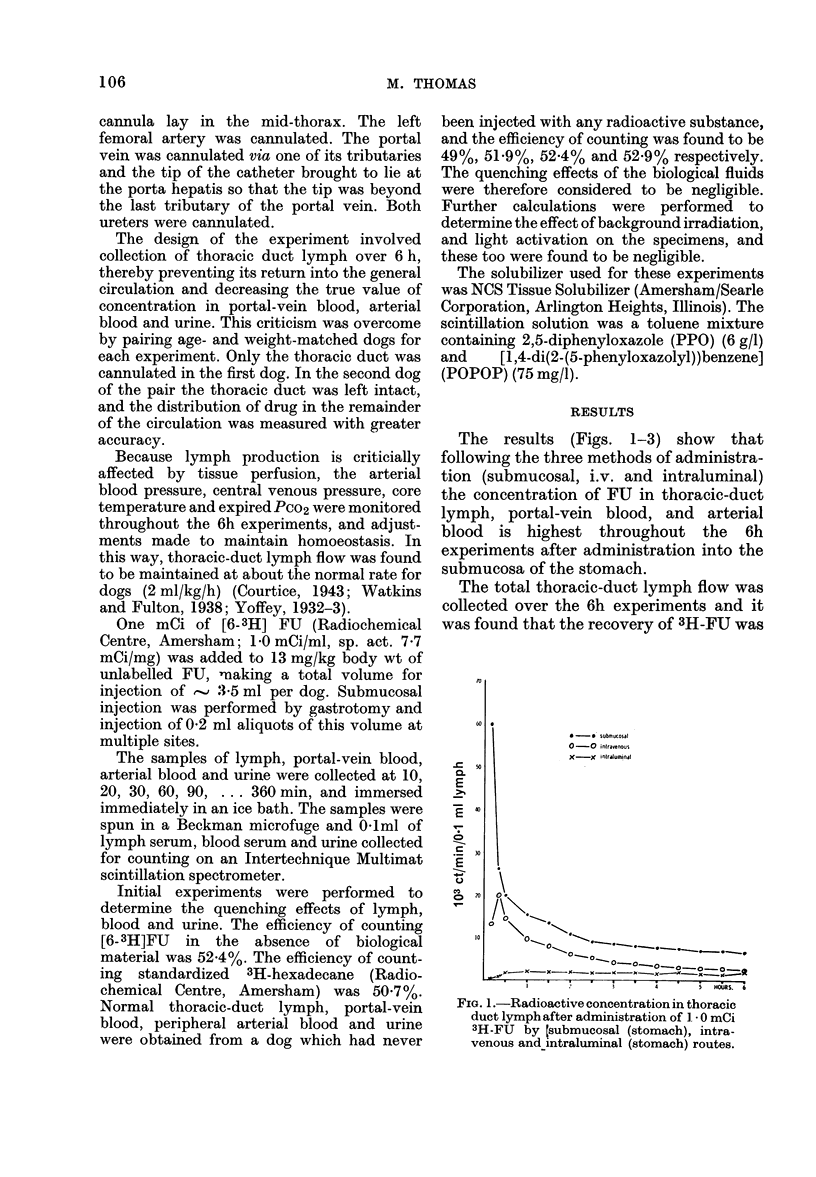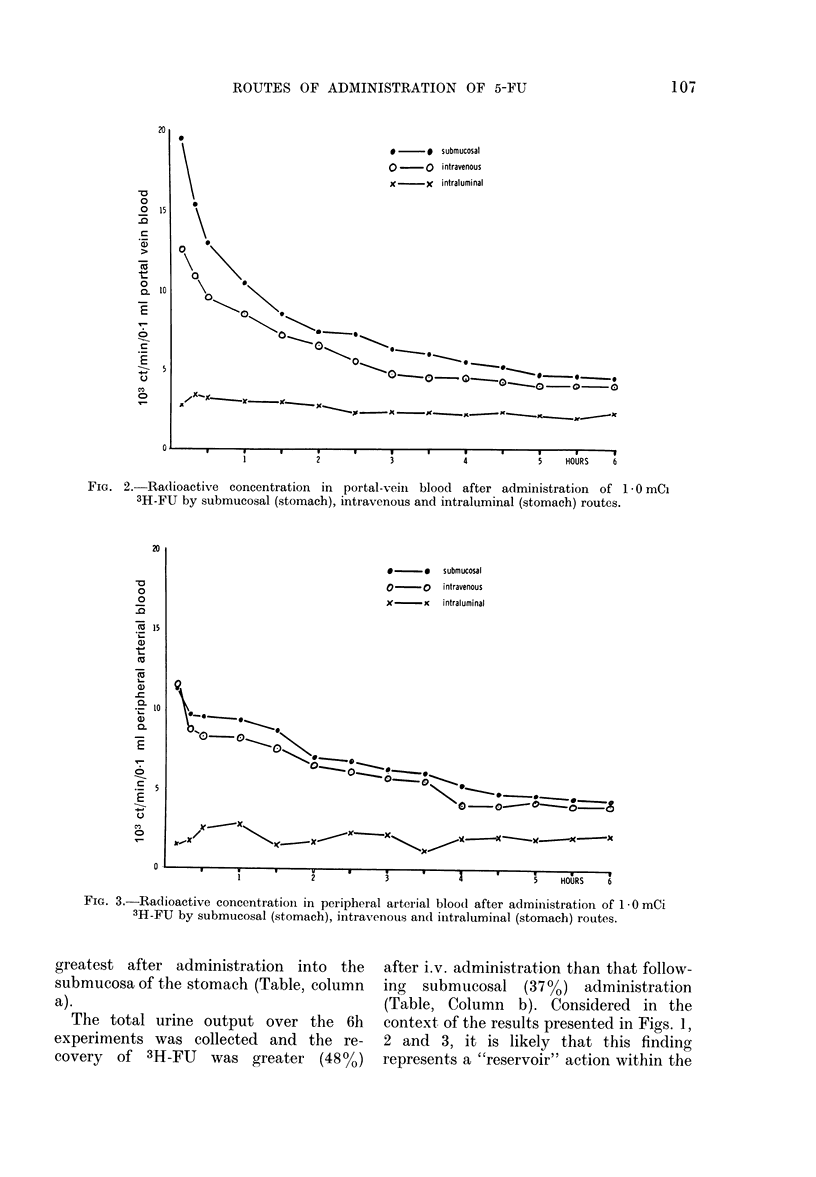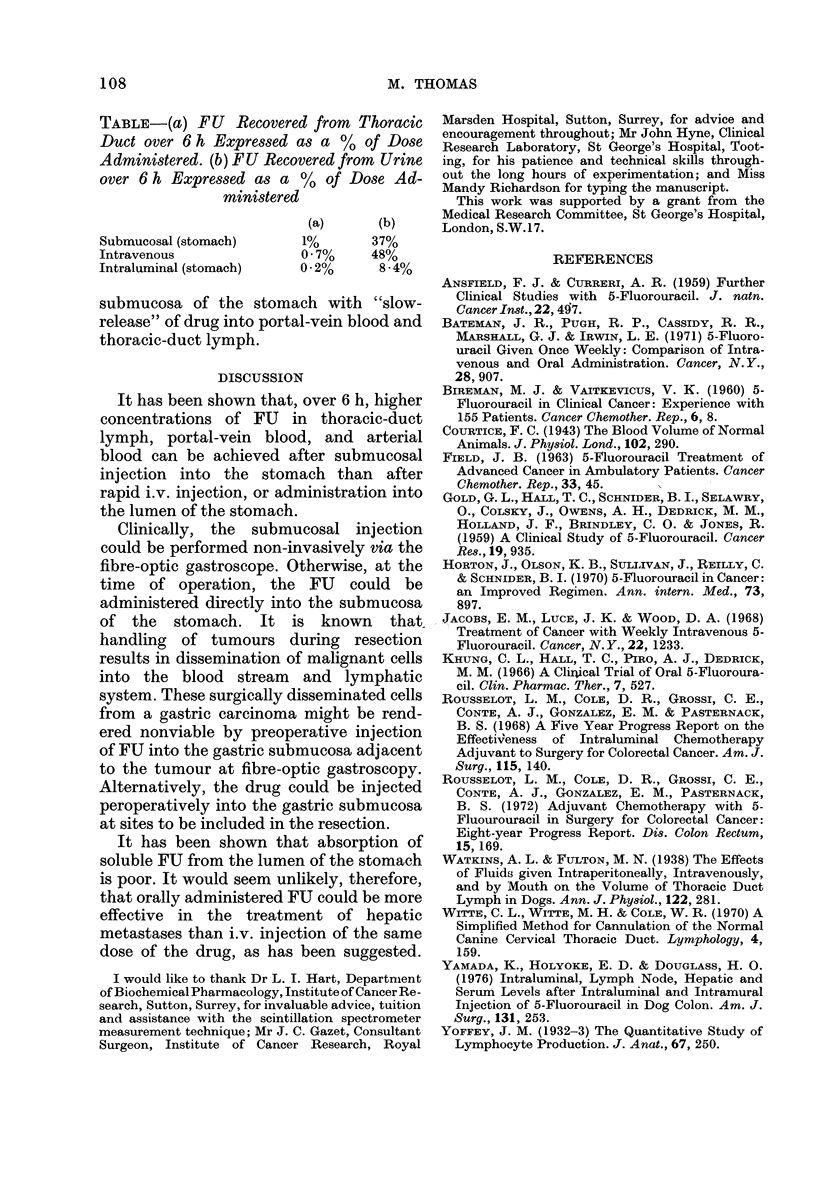# Effect of route of administration of 5-fluorouracil on its concentration in blood and lymph.

**DOI:** 10.1038/bjc.1978.15

**Published:** 1978-01

**Authors:** M. Thomas

## Abstract

The concentration of 5-fluorouracil (FU) in thoracic-duct lymph, portal-vein blood and peripheral arterial blood of beagle dogs was greater after administration into the submucosa of the stomach than after the bolus i.v. injection. The concentration of FU in thoracic-duct lymph, portal-vein blood and arterial blood was least following administration into the lumen of the stomach. The total FU recovered over 6 h from thoracic-duct lymph was compared following the three routes of administration and was found to be greatest following injection into the submucosa of stomach.


*Br. J. Cancer* (1978) **37**, 105.

### EFFECT OF ROUTE OF ADMINISTRATION OF 5-FLUOROURACIL ON ITS CONCENTRATION IN BLOOD AND LYMPH

M. THOMAS

*From the Institute of Cancer Research, Royal Marsden Hospital, Sutton, Surrey*

Received 21 June 1977 Accepted 22 August 1977

**Summary.—The concentration of 5-fluorouracil (FU) in thoracic-duct lymph, portal-vein blood and peripheral arterial blood of beagle dogs was greater after administration into the submucosa of the stomach than after the bolus i.v. injection. The concentration of FU in thoracic-duct lymph, portal-vein blood and arterial blood was least following administration into the lumen of the stomach. The total FU recovered over 6 h from thoracic-duct lymph was compared following the three routes of administration and was found to be greatest following injection into the submucosa of stomach.**

5-Fluorouracil (FU) administered by bolus i.v. injection is an established agent in the palliation of disseminated gastro-intestinal cancer, because of the relatively high response rate (Ansfield and Curreri, 1959; Bireman and Vaitkevicus, 1960; Field, 1963; Gold *et al.*, 1959; Horton *et al.*, 1970; Jacobs, Luce and Wood, 1968).

FU has also been used as an adjuvant to "curative" surgery by peroperative administration into the lumen of the isolated tumour-bearing segment of colon before resection (Rousselot *et al.*, 1968). It was postulated that viable tumour cells in the lumen of the gut would be destroyed, and absorption of the drug into the systemic circulation would destroy malignant cells disseminated by surgical manipulation. An 8-year progress report (Rousselot *et al.*, 1972) describes no improvement in survival of those patients without involvement of mesenteric nodes (American Cancer Society, Stage I and II) but a significantly improved survival among patients with metastatic mesenteric nodes (Stage III). No significant increase in morbidity or mortality was associated with this technique.

It has been suggested (Khung *et al.*, 1966) that orally administered FU may lead to a high concentration in the portal system and be of special benefit to patients with hepatic metastases. Bateman *et al.* (1971) randomized patients with disseminated adenocarcinoma to receive weekly FU either orally or i.v. Clinically useful response was found in 40% of patients receiving oral FU, compared with 21% of patients receiving the drug i.v., but these results were not statistically significant because of small sample size.

Yamada, Holyoke and Douglass (1976) administered FU submucosally into the colon of dogs, and demonstrated a higher concentration in colonic wall, abdominal lymph nodes and liver than that after rapid i.v. injection.

### METHOD

The non-recovery experiments were performed in 6 sex- and weight-matched beagle dogs. Anaesthesia was induced with sodium pentobarbitone and maintained after endotracheal intubation with nitrous oxide and halothane until the necessary cannulations had been performed. Chloralose (100 mg/kg) was then given by rapid i.v. bolus injection to maintain anaesthesia during the 6h experiments.

The thoracic duct was cannulated according to the method of Witte and Witte (1970). The cannula was inserted for several inches into the thoracic duct so that the tip of the cannula lay in the mid-thorax. The left femoral artery was cannulated. The portal vein was cannulated *via* one of its tributaries and the tip of the catheter brought to lie at the porta hepatis so that the tip was beyond the last tributary of the portal vein. Both ureters were cannulated.

The design of the experiment involved collection of thoracic duct lymph over 6 h, thereby preventing its return into the general circulation and decreasing the true value of concentration in portal-vein blood, arterial blood and urine. This criticism was overcome by pairing age- and weight-matched dogs for each experiment. Only the thoracic duct was cannulated in the first dog. In the second dog of the pair the thoracic duct was left intact, and the distribution of drug in the remainder of the circulation was measured with greater accuracy.

Because lymph production is criticially affected by tissue perfusion, the arterial blood pressure, central venous pressure, core temperature and expired $P\text{CO}_2$ were monitored throughout the 6h experiments, and adjustments made to maintain homoeostasis. In this way, thoracic-duct lymph flow was found to be maintained at about the normal rate for dogs (2 ml/kg/h) (Courtice, 1943; Watkins and Fulton, 1938; Yoffey, 1932–3).

One mCi of [6-$^3$H] FU (Radiochemical Centre, Amersham; 1·0 mCi/ml, sp. act. 7·7 mCi/mg) was added to 13 mg/kg body wt of unlabelled FU, making a total volume for injection of ~ 3·5 ml per dog. Submucosal injection was performed by gastrotomy and injection of 0·2 ml aliquots of this volume at multiple sites.

The samples of lymph, portal-vein blood, arterial blood and urine were collected at 10, 20, 30, 60, 90, . . . 360 min, and immersed immediately in an ice bath. The samples were spun in a Beckman microfuge and 0·1ml of lymph serum, blood serum and urine collected for counting on an Intertechnique Multimat scintillation spectrometer.

Initial experiments were performed to determine the quenching effects of lymph, blood and urine. The efficiency of counting [6-$^3$H]FU in the absence of biological material was 52·4%. The efficiency of counting standardized $^3$H-hexadecane (Radiochemical Centre, Amersham) was 50·7%. Normal thoracic-duct lymph, portal-vein blood, peripheral arterial blood and urine were obtained from a dog which had never been injected with any radioactive substance, and the efficiency of counting was found to be 49%, 51·9%, 52·4% and 52·9% respectively. The quenching effects of the biological fluids were therefore considered to be negligible. Further calculations were performed to determine the effect of background irradiation, and light activation on the specimens, and these too were found to be negligible.

The solubilizer used for these experiments was NCS Tissue Solubilizer (Amersham/Searle Corporation, Arlington Heights, Illinois). The scintillation solution was a toluene mixture containing 2,5-diphenyloxazole (PPO) (6 g/l) and [1,4-di(2-(5-phenyloxazolyl))benzene] (POPOP) (75 mg/l).

### RESULTS

The results (Figs. 1–3) show that following the three methods of administration (submucosal, i.v. and intraluminal) the concentration of FU in thoracic-duct lymph, portal-vein blood, and arterial blood is highest throughout the 6h experiments after administration into the submucosa of the stomach.

The total thoracic-duct lymph flow was collected over the 6h experiments and it was found that the recovery of $^3$H-FU was

FIG. 1.—Radioactive concentration in thoracic duct lymph after administration of 1·0 mCi $^3$H-FU by submucosal (stomach), intravenous and intraluminal (stomach) routes.

FIG. 2.—Radioactive concentration in portal-vein blood after administration of 1·0 mCi $^3$H-FU by submucosal (stomach), intravenous and intraluminal (stomach) routes.

FIG. 3.—Radioactive concentration in peripheral arterial blood after administration of 1·0 mCi $^3$H-FU by submucosal (stomach), intravenous and intraluminal (stomach) routes.

greatest after administration into the submucosa of the stomach (Table, column a).

The total urine output over the 6h experiments was collected and the recovery of $^3$H-FU was greater (48%) after i.v. administration than that following submucosal (37%) administration (Table, Column b). Considered in the context of the results presented in Figs. 1, 2 and 3, it is likely that this finding represents a "reservoir" action within the

TABLE—*(a) FU Recovered from Thoracic Duct over 6 h Expressed as a % of Dose Administered. (b) FU Recovered from Urine over 6 h Expressed as a % of Dose Administered*

| | (a) | (b) |
|---|---|---|
| Submucosal (stomach) | 1% | 37% |
| Intravenous | 0·7% | 48% |
| Intraluminal (stomach) | 0·2% | 8·4% |

submucosa of the stomach with "slow-release" of drug into portal-vein blood and thoracic-duct lymph.

### DISCUSSION

It has been shown that, over 6 h, higher concentrations of FU in thoracic-duct lymph, portal-vein blood, and arterial blood can be achieved after submucosal injection into the stomach than after rapid i.v. injection, or administration into the lumen of the stomach.

Clinically, the submucosal injection could be performed non-invasively *via* the fibre-optic gastroscope. Otherwise, at the time of operation, the FU could be administered directly into the submucosa of the stomach. It is known that handling of tumours during resection results in dissemination of malignant cells into the blood stream and lymphatic system. These surgically disseminated cells from a gastric carcinoma might be rendered nonviable by preoperative injection of FU into the gastric submucosa adjacent to the tumour at fibre-optic gastroscopy. Alternatively, the drug could be injected peroperatively into the gastric submucosa at sites to be included in the resection.

It has been shown that absorption of soluble FU from the lumen of the stomach is poor. It would seem unlikely, therefore, that orally administered FU could be more effective in the treatment of hepatic metastases than i.v. injection of the same dose of the drug, as has been suggested.

I would like to thank Dr L. I. Hart, Department of Biochemical Pharmacology, Institute of Cancer Research, Sutton, Surrey, for invaluable advice, tuition and assistance with the scintillation spectrometer measurement technique; Mr J. C. Gazet, Consultant Surgeon, Institute of Cancer Research, Royal Marsden Hospital, Sutton, Surrey, for advice and encouragement throughout; Mr John Hyne, Clinical Research Laboratory, St George's Hospital, Tooting, for his patience and technical skills throughout the long hours of experimentation; and Miss Mandy Richardson for typing the manuscript.

This work was supported by a grant from the Medical Research Committee, St George's Hospital, London, S.W.17.

### REFERENCES

ANSFIELD, F. J. & CURRERI, A. R. (1959) Further Clinical Studies with 5-Fluorouracil. *J. natn. Cancer Inst.*, **22**, 497.

BATEMAN, J. R., PUGH, R. P., CASSIDY, R. R., MARSHALL, G. J. & IRWIN, L. E. (1971) 5-Fluorouracil Given Once Weekly: Comparison of Intravenous and Oral Administration. *Cancer, N.Y.*, **28**, 907.

BIREMAN, M. J. & VAITKEVICUS, V. K. (1960) 5-Fluorouracil in Clinical Cancer: Experience with 155 Patients. *Cancer Chemother. Rep.*, **6**, 8.

COURTICE, F. C. (1943) The Blood Volume of Normal Animals. *J. Physiol. Lond.*, **102**, 290.

FIELD, J. B. (1963) 5-Fluorouracil Treatment of Advanced Cancer in Ambulatory Patients. *Cancer Chemother. Rep.*, **33**, 45.

GOLD, G. L., HALL, T. C., SCHNIDER, B. I., SELAWRY, O., COLSKY, J., OWENS, A. H., DEDRICK, M. M., HOLLAND, J. F., BRINDLEY, C. O. & JONES, R. (1959) A Clinical Study of 5-Fluorouracil. *Cancer Res.*, **19**, 935.

HORTON, J., OLSON, K. B., SULLIVAN, J., REILLY, C. & SCHNIDER, B. I. (1970) 5-Fluorouracil in Cancer: an Improved Regimen. *Ann. intern. Med.*, **73**, 897.

JACOBS, E. M., LUCE, J. K. & WOOD, D. A. (1968) Treatment of Cancer with Weekly Intravenous 5-Fluorouracil. *Cancer, N.Y.*, **22**, 1233.

KHUNG, C. L., HALL, T. C., PIRO, A. J., DEDRICK, M. M. (1966) A Clinical Trial of Oral 5-Fluorouracil. *Clin. Pharmac. Ther.*, **7**, 527.

ROUSSELOT, L. M., COLE, D. R., GROSSI, C. E., CONTE, A. J., GONZALEZ, E. M. & PASTERNACK, B. S. (1968) A Five Year Progress Report on the Effectiveness of Intraluminal Chemotherapy Adjuvant to Surgery for Colorectal Cancer. *Am. J. Surg.*, **115**, 140.

ROUSSELOT, L. M., COLE, D. R., GROSSI, C. E., CONTE, A. J., GONZALEZ, E. M., PASTERNACK, B. S. (1972) Adjuvant Chemotherapy with 5-Fluourouracil in Surgery for Colorectal Cancer: Eight-year Progress Report. *Dis. Colon Rectum*, **15**, 169.

WATKINS, A. L. & FULTON, M. N. (1938) The Effects of Fluids given Intraperitoneally, Intravenously, and by Mouth on the Volume of Thoracic Duct Lymph in Dogs. *Ann. J. Physiol.*, **122**, 281.

WITTE, C. L., WITTE, M. H. & COLE, W. R. (1970) A Simplified Method for Cannulation of the Normal Canine Cervical Thoracic Duct. *Lymphology*, **4**, 159.

YAMADA, K., HOLYOKE, E. D. & DOUGLASS, H. O. (1976) Intraluminal, Lymph Node, Hepatic and Serum Levels after Intraluminal and Intramural Injection of 5-Fluorouracil in Dog Colon. *Am. J. Surg.*, **131**, 253.

YOFFEY, J. M. (1932–3) The Quantitative Study of Lymphocyte Production. *J. Anat.*, **67**, 250.